# The Utility of Serial Echocardiography Parameters in Management of Newborns with Congenital Diaphragmatic Hernia (CDH) and Predictors of Mortality

**DOI:** 10.1007/s00246-022-03002-y

**Published:** 2022-09-27

**Authors:** Roopali Soni, Naharmal Soni, Aravanan Chakkarapani, Samir Gupta, Phani Kiran Yajamanyam, Sanoj K. M. Ali, Mohammed El Anbari, Moath Alhamad, Dhullipala Anand, Kiran More

**Affiliations:** 1 Division of Neonatology, Sidra Medicine, Al Luqta Street, Education City North Campus, Qatar Foundation, Doha, Qatar; 2 Weill Cornell Medicine, Al-Rayyan, Doha, Qatar; 3 Neonatal Unit, Mediclinic Parkview Hospital, Dubai, UAE

**Keywords:** Congenital diaphragmatic hernia, Pulmonary hypertension, Functional echocardiography, Cardiac dysfunction, Mortality

## Abstract

Ventricular dysfunction may be found in 40% of newborns with CDH, and is not only a predictor of disease severity, but also mortality and need for ECMO. We conducted this study to assess the utility of serial echocardiography in management of newborns with CDH and their survival outcomes. This is a retrospective study, wherein the demographic, clinical and echocardiographic data from our local CDH registry and hospital clinical database were analyzed to study the correlation of timed echocardiographic findings with mortality and other outcomes. Fourty-two newborns with CDH were admitted during the study period (M/F:19/23), with median gestation of 38 weeks (IQR:36–39) and birth weight of 2.83 kg (IQR 2.45–3.17). Thirty-one were left-sided, seven right, one central, and three bilateral hernias. Twelve infants (28%) died in early infancy. Three infants were excluded from analysis due to either palliation at birth or significant cardiac anomaly. A total of 137 echos from 39 infants were analyzed. Seventy percent of newborns who died and had an echo within the first 72 h, were noted to have suffered from moderate to severe PH. Birth weight < 2.8 kg, RVSP > 45.5 in the first 72 h and postoperative VIS > 23.5 and RSS > 4.3 were good predictors of mortality. Markers of elevated pulmonary pressures and cardiac function were useful in guiding therapy. Serial timed functional echocardiography (f-Echo) monitoring allows targeted therapy of patients with CDH. Birth weight, initial severity of pulmonary hypertension and postoperative RSS and VIS may be useful in predicting mortality.

**Supplementary Information:**

The online version contains supplementary material available at 10.1007/s00246-022-03002-y.

## Background

Congenital diaphragmatic hernia (CDH) is a life-threatening defect in the developing diaphragm's integrity [1], with a reported incidence of less than 3 per 10,000 live births [2], and is often accompanied by other congenital anomalies. Management of newborn infants with CDH requires a high skill set, with multidisciplinary team involvement, starting from the point of antenatal diagnosis. Despite improved outcomes over the years [3], morbidity and mortality remain high (20–40%), even in high volume tertiary referral centers [4, 5]. A quarter of survivors suffer neurodevelopmental impairment involving all domains, and ranges from motor and sensory (hearing, visual) deficits to cognitive, language, and behavioural impairment [6]. Various clinical and laboratory parameters and prognostic indices in the perinatal period have been studied to help predict outcomes and mortality [5, 7–10]. Standardized management and regionalized care, involving complex ventilatory and hemodynamic management, improve survival outcomes [11]. Although the need for Extracorporeal membrane oxygenation (ECMO) [12] in CDH patients has decreased over the years due to improved understanding of physiology and advances in prenatal and neonatal care, CDH still remains in the top three indications for neonatal respiratory ECMO [13], and mortality in this group of CDH patients remains unchanged [14, 15].

Patients with CDH have varying degrees of pulmonary hypoplasia and abnormal pulmonary vascular disease, often leading to various degrees of pulmonary hypertension. In CDH patients, persistent pulmonary hypertension of the newborn (PPHN) is associated with adverse outcomes, and therefore its management remains crucial in the care of these infants [16]. Up to 30–40% of newborns with CDH can have left (LV) or right ventricular (RV) or biventricular dysfunction, and ventricular interdependence may play a role [17, 18] Association of ventricular dysfunction and ventricular performance is not only a predictor for disease severity [19], but also for mortality [20] and need for ECMO [21].

Advances in our understanding of cardiovascular physiology in CDH patients has allowed for the optimization of hemodynamics and management of pulmonary hypertension (PH) [22]. Timed functional echocardiography (f-Echo) assessment, with predefined criteria for assessing the degree of pulmonary hypertension and ventricular dysfunction, helps in the effective management of newborns with CDH. This also aids decision-making in the timing of CDH repair and managing acute postoperative clinical decompensation from pulmonary hypertensive crisis [23].

Non-response to pulmonary arterial vasodilators in CDH patients may be related to abnormal ventricular function, and it is important to improve cardiac function in addition to reducing pulmonary arterial pressures in this situation [24].

Sidra Medicine is a greenfield children’s hospital in Doha, Qatar. The neonatal intensive care unit (NICU) is a quaternary referral center for national and international patients with medical, surgical, and cardiac conditions. A national CDH-Qatar (CDH-Q) program was established and standard clinical guidelines for the management of CDH were developed based on recent literature and international guidelines. This study was conducted at Sidra Medicine to test the hypothesis that routine serial echocardiography is an important tool in the standardized management of newborns with CDH, and may improve survival outcomes.

## Methods

### Study Design

This is a retrospective, observational, single center study conducted at Sidra Medicine hospital, from April 2018 to January 2022. A retrospective review of echocardiography images and clinical charts of all eligible CDH patients admitted to the NICU was done using the hospital's electronic medical record system.

### Setting

The NICU at Sidra Medicine hospital has facilities for ECMO, and is well supported by a neonatal hemodynamics team of consultant neonatologists, with expertise in functional echocardiograph. The CDH-Qatar (CDH-Q) program was established with a vision to develop a national quaternary referral center for pre-and post-natal referrals of CDH patients in Qatar and further afield. A local CDH registry was created at Sidra Medicine in 2019 on REDCap® (Research Electronic Data Capture) software [25] for data sharing and reporting to international CDH registries[26]. The department used an f-Echo protocol, adapted from the CoDiNOS trial [27] by the CDH EURO Consortium, for serial assessment of myocardial function and PPHN in CDH patients, at set time points, in order to guide cardiorespiratory management, surgical intervention, and follow up care pre- and post-discharge. Members of hemodynamic and ECMO teams closely followed patients to minimize variations in clinical practice and assist in f-Echo based decisions. Following on from the establishment of CDH-Q program and foundation of standardized management practices (Appendix I), we conducted this retrospective review, with the below objectives.

Primary Objectives:

1. To assess the impact of standardized management, utilizing serial echocardiography, on mortality amongst newborns with CDH.
2. To identify echocardiography predictors of mortality.

Secondary Objectives:

1. To study the correlation of echocardiography markers with respiratory severity score (RSS) and inotropic scores in predicting disease severity and mortality.
2. To evaluate trends in f-echo and cardiorespiratory parameters used in decision-making for timing of surgical correction.
3. To study the use of inotropes and milrinone in the treatment of ventricular dysfunction and various pulmonary vasodilators in treatment of acute or chronic pulmonary hypertension related to CDH.

### Participants

- *Inclusion Criteria* All infants admitted to Sidra NICU with a diagnosis of CDH from April 2018 till January 2022.
- *Exclusion Criteria* Infants who received palliative care from birth and those with significant associated congenital cardiac anomalies, other than atrial septal defect (ASD), ventricular septal defect (VSD), and persistent ductus arteriosus (PDA), were excluded from f-Echo analysis but were included in the basic demographic data analysis.

### Data Collection

Study data were collected and managed using secure, web-based software-REDCap® electronic data capture tools hosted at Sidra Medicine, Qatar. Patient demographic data included infants’ sex, gestational age, birth weight, prenatal MRI and ultrasound scan results, postnatal treatment, side and size of the defect, age at repair, cardiovascular therapies, ECMO, duration of ventilation, and non-invasive ventilatory support. For size of CDH defect, Congenital Diaphragmatic Hernia Study Group (CDHSG) Staging System was utilized [28]. In addition, the outcome data on death, discharge, and length of stay was extracted from the CDH registry. Any missing data were collected from the electronic medical records on Cerner Powerchart® (North Kansas City, US) patient management software. Established ECHO protocols in the unit for assessment of myocardial function and PPHN (Appendix IIA), and standardized timings for these assessments were used to collect data (Appendix IIB).

All echocardiograms were performed using the EPIC 7 Philips machine with a S12-4 Hz/ S8-3 multi-frequency sector probe. The first, structural scans, were performed by pediatric cardiology. Functional echocardiography scans were thereafter performed by clinicians from the neonatal hemodynamics team. Researchers collected echocardiography data from the Philips Intellivue® database. Two researchers measured some of the missing f-Echo parameters offline, wherever possible, and cross-verified them to minimize inter-observer variability in reporting.

### Timeline for f-Echos

A total of three echocardiograms was studied for each patient at the set time points:

- within 72 h of birth
- 24–48 h pre-operative
- 24–48 h post-operative

### ECHO Parameters

A standard Echo protocol as per published guidelines was followed, and all possible functional Echo parameters were collected as listed in Appendix IIA.

The following measurements were taken:

#### Persistent Pulmonary Hypertension of the Newborn (PPHN)

PPHN was diagnosed by assessment of right ventricular systolic pressure (RVSP) [calculated from tricuspid regurgitant jet velocity, using the Bernoulli equation], the presence of right-to-left or bidirectional shunting at atrial level or ductus arteriosus, septal wall flattening or paradoxical motion. PH was defined as moderate if RVSP ≥ 45, with or without bidirectional /right-to-left ductal shunting and severe if RVSP ≥ 70 and bidirectional /right-to-left ductal shunting [29, 30] Pulmonary vascular resistance (PVR) index was calculated as right ventricular ejection time (RVET)/pulmonary artery time-to-peak velocity (TPV).

#### Cardiac Function (See Supplementary Material)

1. Markers of LV function – systolic function is assessed by fractional shortening (FS), ejection fraction (EF) of LV with the biplane Simpson's method, and left ventricular output (LVO). Markers of LV diastolic function are early to late ventricular filling velocities (E/A ratio) and isovolumic relaxation time (IVRT) [31] The mean of three cardiac cycles was calculated for all hemodynamic parameters and used for further analysis and calculations as per standard protocol [31] Left ventricular systolic dysfunction was defined by fractional shortening less than 25% or ejection fraction less than 50%.
2. Right ventricular systolic function was assessed using Tricuspid Annular Plane Systolic Excursion (TAPSE) and Fractional Area Change (FAC).

TAPSE was evaluated by measuring total excursion of tricuspid annulus during ventricular systole on M-mode using apical four-chamber view with cursor aligned in the direction of movement of tricuspid annulus. Three measurements were taken, and an average value at two decimal points was recorded for analysis.

FAC was measured using the 2D recording of RV in a four-chamber view by measuring RV area in systole and diastole.

#### Cardiorespiratory Parameters

Relevant cardiorespiratory parameters were collected at the time of Echo at corresponding time points.

Respiratory severity score (RSS) was calculated using mean airway pressure (MAP) x fractional inspired oxygen (FiO2), and was considered as a marker of severity of illness [32]. Subgroup analysis was done comparing newborns with RSS less than 4 versus more than 4 as a severity cut-off used in previously published studies [32].

Vasoactive Inotropic score (VIS) was calculated using the following formula [33]:

VIS = (dopamine dose (μg/kg/min) + dobutamine dose (μg/kg/min) + 100 × epinephrine dose (μg/kg/min) + 10 × milrinone dose (μg/kg/min) + 10 000 × vasopressin dose (unit/kg/min) + 100 × norepinephrine dose (μg/kg/min).

Data were also collected for inhaled nitric oxide (iNO) and other pulmonary vasodilators, alprostadil and hydrocortisone.

The following outcome data were recorded from Cerner and REDCap®-death, need for ECMO, duration of ventilation, respiratory support or oxygen, inhaled nitric oxide (iNO), other pulmonary vasodilators, various types of inotropic support, day of CDH surgical repair, and length of hospital stay.

Institutional Review Board (IRB) approval for this project was obtained from Sidra Medicine (IRB No-1696088-4). STROBE criteria were followed for reporting this observational study (see Supplementary Material).

### Statistical Analysis

Descriptive statistics were used to describe the study population, including gender, age (in days), gestation at birth, birth weight, and singleton/multiple births. Categorical and continuous variables were represented by frequencies, mean ± SD or median (Inter quartile range). To compare continuous variables between groups, the Mann–Whitney *U*-test was used. For calculating the p-values of association between categorical variables, the Chi-square or Fisher's Exact test was used as appropriate. Freeman's coefficient of differentiation was used as a measure of association between defect size and mortality where 0 represents no association and 1 represents perfect association. Serial TAPSE measurements were analyzed using the rstatix package and a one-way repeated measures ANOVA, which is an extension of the paired-samples t-test for comparing the means of three or more levels of the within-subjects variable. To build ROC curves, the pROC package was used. The Youden Index was used to determine the appropriate cut-off point for the parameters of interest. Survival Analysis was performed using the Kaplan–Meier Model. All statistical analyses were performed using the statistical software R version 4.1.1. Any association with a p-value less than 0.05 was considered significant.

## Results

### Participants

Fourty-two newborns with CDH were admitted during the specified study period (M/F:24/18), with median gestation age of 38 weeks (IQR:36–39) and median birth weight of 2.83 kg (IQR 2.45–3.17). Thirty-one were left-sided, 7 right-sided, 1 central and 3 bilateral diaphragmatic hernias. Three patients had D type and 9 had C type defect, 20 had either A or B defects. Information on defect size was unavailable in 10 patients, where they were either not operated upon or the surgeon had not clearly stated the size of defect. Descriptive data were collected for all 42 infants, but three infants were excluded from analysis, as per exclusion criteria, due to either palliation at birth or significant cardiac anomaly—one infant with transposition of great arteries (TGA) and ASD, VSD, hypoplastic tricuspid valve and another with TGA and atrioventricular septal defect.

### Mortality Data

A total of 12 out of 42 infants admitted with CDH died in NICU/CICU, of which 5 had abnormal genetics. Our overall mortality rate was 28%, and after excluding genetic anomalies was 19%. Mortality amongst preterm infants (< 37 weeks gestation) was 45%; that in term infants was 22%. A total of 37 infants underwent surgical repair for CDH, out of which 3 died within 30 days and 4 more died at later stages. Of the 2 patients that required ECMO, 1 survived. Both the infants with significant heart anomalies died (one was palliated from birth and the other died post hernia repair). Mortality amongst infants with milder congenital heart defects was 33%.

Demographic data was compared based on survivors and non-survivors; no significant difference was found amongst these two groups, apart from side of defect, where those with bilateral hernias had a higher mortality (Table 1). Clinical characteristics and associated conditions in babies who didn’t survive is given in Table 2.

**Table 1:** Demographic characters of infants

	No mortality (*n* = 30)	Mortality (*n* = 12)	*p*-value
Comparison of Demographic and clinical characteristics of infants with CDH based on outcome of mortality			
Gestational age (weeks),^a^	37.7 (2.88)	36.1 (2.35)	NS
Birth weight (grams)^a^	2.17 (0.82)	2.30 (0.72)	NS
Gender (male%)	12 (40%)	6 (50%)	NS
Genetic Anomalies (N %)	5 (16.7%)	5 (41.7%)	NS
CDH side	Left 24 (77%) Right 6 (20%) Bilateral 0	Left 8 (67%) Right 1 (8.3%) Bilateral 3 (25%)	0.035
CDH size^c^	5A, 12B, 7C, 2D	1A, 2B, 2C, 1D	NS
Age at surgery (days of life);^a^	8.7 (8.6)	7.6 (6.7)	NS
Pre-operative RSS^a^	4.42 (4.61)	3.55 (4.69)	NS
Post-operative RSS^a^	3.70 (2.85)	5.66 (6.71)	NS
Pre-operative inotropic use (VIS^b^)^a^	3.20 (6.13)	6.08 (11.2)	NS
Milrinone treatment Post-op (n %)	10 (33.3%)	5 (41.7%)	NS

^a^Mean (SD)

^b^Vasoactive Ionotropic score(VIS): (dopamine dose (μg/kg/min) + dobutamine dose (μg/kg/min) + 100 × epinephrine dose (μg/kg/min) + 10 × milrinone dose (μg/kg/min) + 10 000 × vasopressin dose (unit/kg/min) + 100 × norepinephrine dose (μg/kg/min)

^c^CDH size as per Congenital Diaphragmatic Hernia Study Group (CDHSG) Staging System

**Table 2:** Clinical characteristics and comorbidities of newborn infants with mortality

No	Birth gestation	Gender	Birth weight (Kg)	Side of defect	Size of defect	Age at death(days)	Comorbidity
1	37	F	1.75	Bilateral	N/A	5 (pre-repair)	IUGR, Genetic anomaly
2	38	M	3.12	Bilateral	N/A	1 (pre-repair)	Lung hypoplasia, bilateral pneumothoraces
3	32	F	1.4	Left	ND	95	Premature, Mosaic trisomy 1q anomaly, seizures, infections
4	36	M	2.76	Right	C	16 (pre-repair)	Premature, Postnatal diagnosis and referral for ECMO on Day 1
5	32	F	1.58	Bilateral	N/A	1 (pre-repair)	Palliated at birth, premature, severe lethal pulmonary hypoplasia
6	38	F	2.45	Left	A	25	Trisomy 13
7	35	M	2.22	Left	C	341	Premature, Genetic defect, Bronchomalacia, large VSD, ASD
8	38	F	2.5	Left	ND	90	Genetic defect, TGA, arch hypoplasia
9	34	M	1.63	Left	D	231	Premature, Severe rebound pulmonary hypertension, tracheostomy, ASD
10	37	M	2.1	Left	N/A	1 (pre-repair)	Palliated at birth, TGA, AVSD, Pulmonary atresia
11	37	M	3.77	Left	B	22	Twisting of ileo-caecal valve-colostomy
12	39	F	2.97	Left	B	10	Bowel necrosis, 50 cm of bowel resected

Total of ten infants had abnormal genetics, out of which six infants had identifiable genetic syndromes [Trisomy 13, Mosaic trisomy of 1q, Congenital myasthenia gravis, Phelan-McDermid syndrome, Wolf-Hirschhorn Syndrome (WHS), Chromosome 12p duplication (Pallister-Killian Syndrome)]

### Outcomes Data

#### Primary Outcomes

##### Echocardiography-based Management of CDH Patients

A total of 137 echocardiograms in 39 newborns were analyzed, where available, at the specified time intervals. A review of patient scans and notes confirmed that serial echocardiography findings were consistently used in all patients to influence decisions regarding initiation and choice of inotropes and use of milrinone, inhaled nitric oxide, sildenafil, inhaled iloprost, vasopressin, alprostadil. ECHO markers of moderate to severe pulmonary hypertension (RVSP, bidirectional shunting, TPV/RVET ratio) influenced use of pulmonary vasodilators like inhaled nitric oxide (iNO) and IV/oral sildenafil. INO was mainly used if RVSP > 60 mm Hg and FiO2 requirement > 60% either during initial stabilisation, perioperative period or during episodes of acute deterioration over chronic pulmonary hypertension (PH).

Subnormal TAPSE with or without low RVO/LVO were particularly useful in guiding use of Milrinone alone or with epinephrine. However, no statistical correlation could be made with these outcome data. Fluid boluses were used with caution for intravascular fluid depletion, upon ECHO guided assessment.

In addition, echocardiography was used to influence discussions and decisions regarding initiation of ECMO, readiness for surgery and pre-and post-operative stabilization. Seventy percent (7/10) of the newborns that died and had serial echo assessments, were found to have suffered from moderate to severe PH in the first 72 h.

##### Predictors of Mortality

We studied the utility of a number of demographic characteristics, RSS and f-Echo parameters with regards to predicting mortality.

Correlation of defect size with mortality was analyzed. A value of 0.16 was obtained for Freeman's coefficient of differentiation between defect size and mortality, indicating no association.

**Fig. 1:**
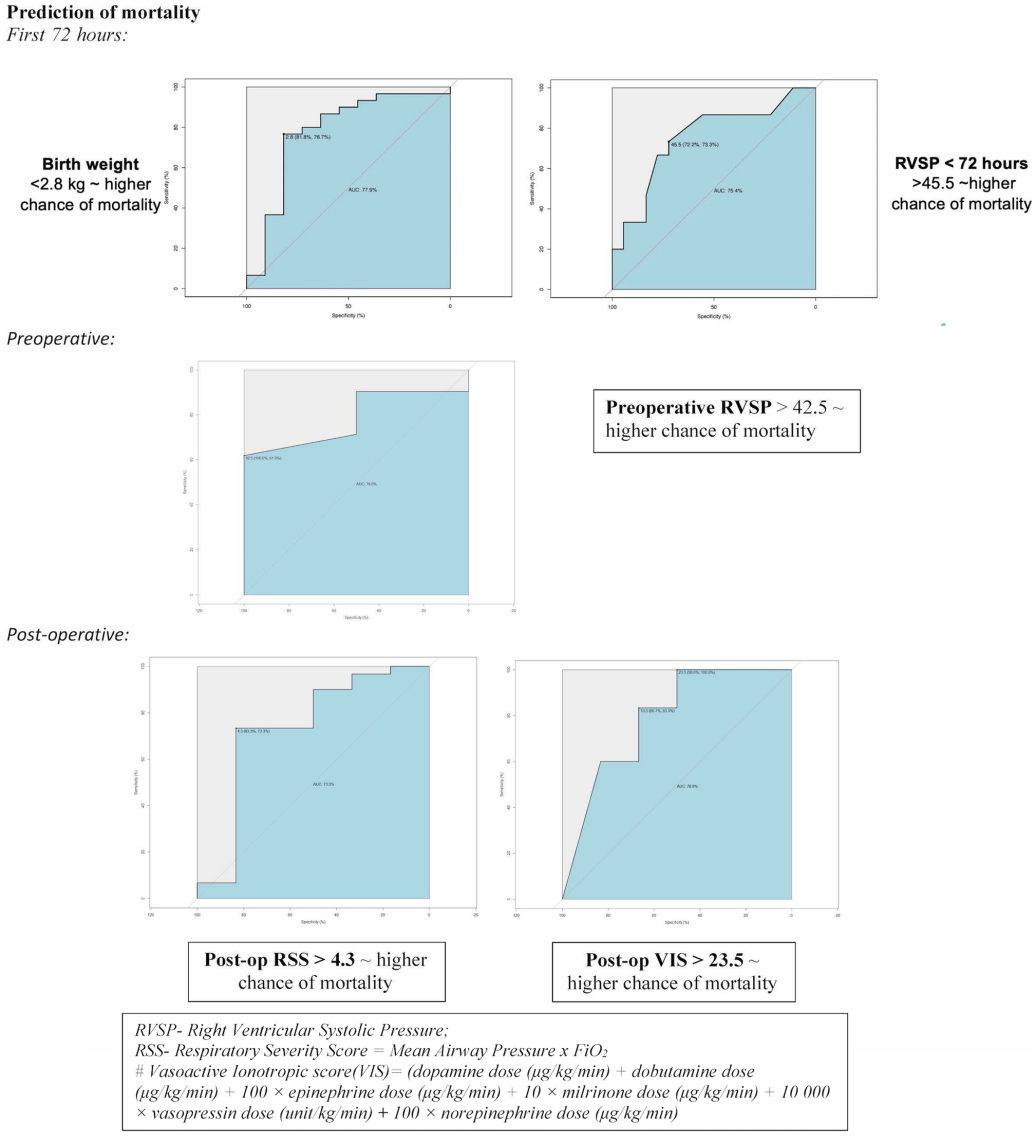
Clinical and ECHO parameters predicting mortality

Receiver Operating Curve (ROC) curves were used for analyzing all other parameters (Fig. 1). The statistically significant predictors were:

Birth weight less than 2.8 kg (specificity of 81.8% and sensitivity of 76.7%) was identified to be a significant marker in predicting mortality with AUC of 77.9%.

RVSP of more than 45.5 (72.2%, 73.3%) in the first 72 h, suggesting moderate pulmonary hypertension, was a good predictor of mortality with AUC of 75.4%, this cut-off value was found to be only slightly lower in the pre-operative period, at 42.5 (100%, 62%; AUC ~ 78.6).

VIS in post-operative period, showed two significant values that were predictive of mortality (calculated using the Youden Index). With AUC of 78.9%, VIS of 10.5 had a sensitivity of 83.3% and specificity of 66.7% and VIS of 23.5 was 100% sensitive but only 50% specific.

RSS of ≥ 4.3 in the postoperative period (AUC 73.3%) was predictive of mortality with a specificity of 83.3% and sensitivity of 73.3%.

**Fig. 2:**
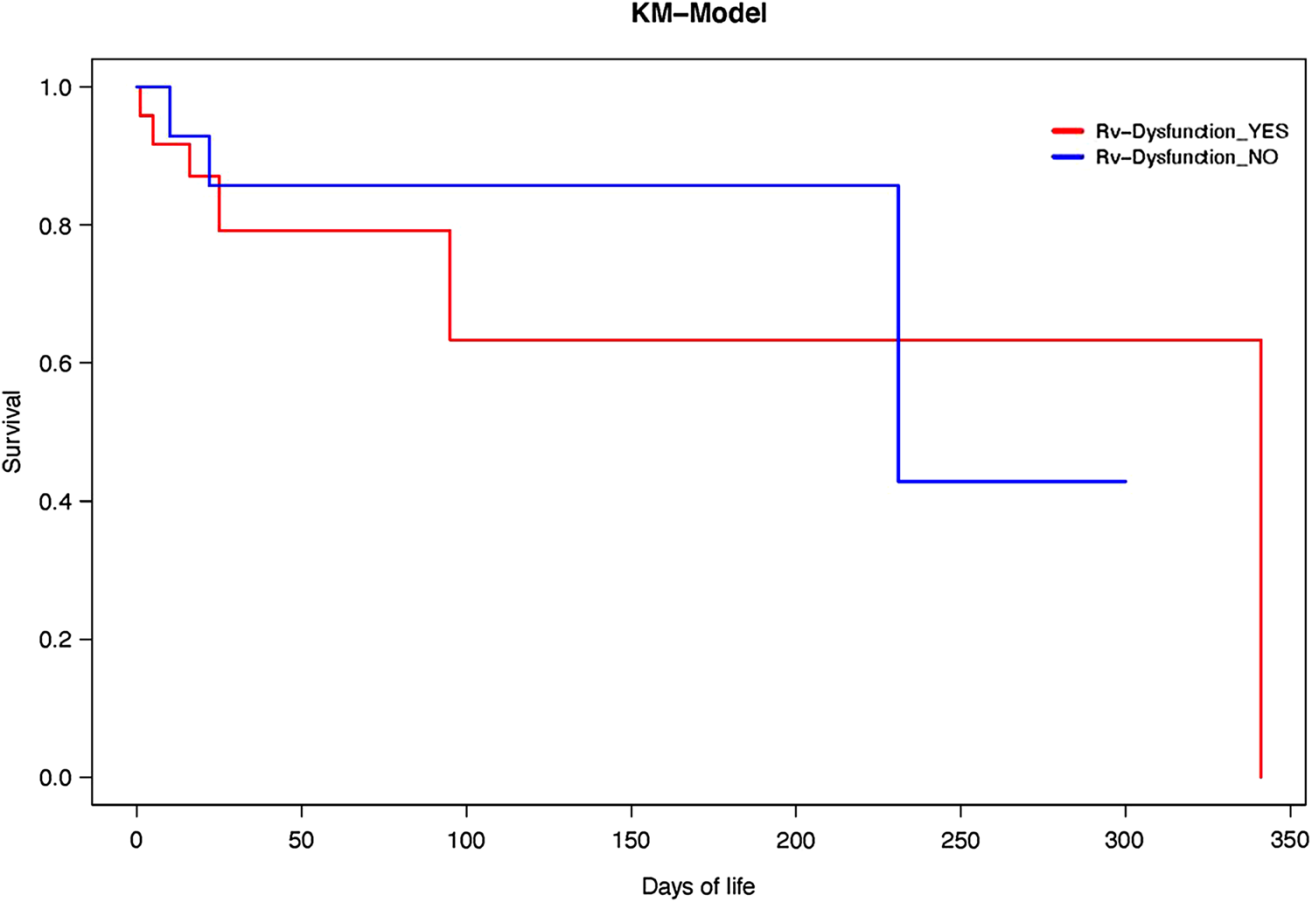
Effect of RV dysfunction on mortality

Mortality prediction based on RV dysfunction:

Kaplan–Meier plot of all the patients showed increased trends towards early mortality in those having RV dysfunction compared to those who didn’t; however, this was not statistically significant (Fig. 2).

#### Secondary Outcomes

##### Correlation of Echocardiography Markers with RSS and VIS

We compared the echo parameters of babies with low respiratory severity scores (RSS < 4) with those of higher scores (RSS > 4) and found significant correlation with certain echo parameters in first 72 h, pre- and post-op (Table 3). Overall, this suggests that those with higher RSS had higher pulmonary pressures and higher use of inotropes.

**Table 3:** Comparison of relevant ECHO parameters based on RSS

	RSS < 4	RSS ≥ 4	*p*-value
First 72 h	(*n* = 22)	(*n* = 17)	
Birth GA; mean (SD)	36.9 (3.24)	38.0 (1.97)	0.19
Birth Weight; mean (SD)	2.74 (0.77)	2.74 (0.77)	0.318
RVSP; mean (SD)	35.8 (20.5)	53.7 (20.9)	0.019
RV Dysfunction (n%)	1(4.5%)	10 (58%)	0.029
PDA shunting (n%)	Left to Right = 7 (32%) Bidirectional = 10 (45%) Right to left = 0	Left to Right = 3 (17.6%) Bidirectional = 8 (47%) Right to left = 4 (23.5%)	0.07
PFO shunting (n%)	Bidirectional = 1 (4.5%) Right to left = 3 (13.6%)	Bidirectional = 6 (35%) Right to left = 2 (11.8%)	0.002
Milrinone (%)	2 (9%)	16 (94%)	< 0.001
Inotropic score; mean (SD)	0.50 (1.34)	17.2 (10.4)	< 0.001
Pre-operative	(*n* = 25)	(*n* = 11)	
RVSP; mean (SD)	41.0 (16.8%)	43.8 (30.8%)	NS
PDA shunting (R to L,or bidirectional) (n%)	3 (13.6%)	4 (23.5%)	0.08
PFO shunting (bidirectional)	0	4 (36%)	0.009
Milrinone (n%)	9 (36.0%)	9 (36.0%)	0.022
Inotropic score; mean (SD)	1.97 (5.30)	8.00 (8.26)	0.052
Inhaled Nitric oxide (NO) (n%)	4 (16%)	6 (54%)	0.017
Post-operative	(*n* = 23)	(*n* = 13)	
RVSP; mean (SD)	31.2 (29.3)	69.2 (27.4)	0.074
PDA shunting (R to L, or bidirectional) (n%)	0	5 (38.4%)	0.034
Severity of PH (Moderate or severe) (n%)	1 (4.3%)	5 (38.4%)	0.048
Milrinone (n%)	6 (26%)	12 (92%)	0.001
Inotropic score; mean (SD)	2.26 (5.2)	14.2 (9.6)	0.001
Inhaled Nitric oxide (NO)	3 (13%)	9 (69%)	0.0006

*RVSP* Right Ventricular Systolic Pressure, *PDA* Patent Ductus Arteriosus, *PFO* Patent formaen Ovale, *IVS* Interventricular septum

We also compared the Echo parameters for babies with lower inotropic scores (VIS < 15) versus higher scores (VIS ≥ 15) [34]. There was significant difference in some of the Echo parameters (Table 4) suggesting more severe pulmonary hypertension and cardiac dysfunction in the high VIS group.

**Table 4:** Comparison of ECHO parameters based on VIS Score (< 15 vs ≥ 15)

Inotropic Score (VIS)	VIS Score (< 15)	VIS Score ≥ 15	*p*-value
First 72 h	(*n* = 29)	(*n* = 10)	
Birth GA; mean (SD)	37.1 (2.94)	37.1 (2.94)	0.16
Birth Weight; mean (SD)	2.76 (0.71)	3.01 (0.61)	0.3
RVSP; mean (SD)	35.6 (19.6)	63 (15.5)	< 0.001
RV Dysfunction (n%)	7 (25%)	7 (70%)	0.021
TAPSE	8.28 (1.4)	8 (1)	NS
PDA shunting (n%)	Left to Right = 10 (42%) Bidirectional = 13 (54%) Right to left = 0	Left to Right = 0 Bidirectional = 5 (55%) Right to left = 4 (44%)	0.001
PFO shunting (n%)	Left to Right = 20(91%) Bidirectional = 2 (9%) Right to left = 0	Left to Right = 2 (22.2%) Bidirectional = 5 (55.6%) Right to left = 4 (44.4%)	< 0.001
Severity of PH (Moderate or severe) (n%)	13 (45%)	10 (100%)	0.001
Milrinone (%)	8 (27%)	10 (100%)	< 0.001
Pre-operative	(*n* = 33)	(*n* = 3)	
RVSP; mean (SD)	41.8 (22.3)	43.3 (24.7)	NS
PDA shunting (R to L,or bidirectional) (n%)	6 (40%)	1 (33%)	NS
PFO shunting (bidirectional)	3 (19%)	1 (33%)	NS
Severity of PH (Moderate or severe) (n%)	14 (60%)	3 (100%)	NS
Milrinone (n%)	16 (49.0%)	3 (100%)	0.022
Inhaled Nitric oxide (NO) (n%)	7 (21%)	3 (100%)	0.017
Post-operative	(*n* = 23)	(*n* = 13)	
RVSP; mean (SD)	31.2 (29.3)	69.2 (27.4)	0.074
PDA shunting (R to L, or bidirectional) (n%)	0	5 (38.4%)	0.034
Severity of PH (Moderate or severe) (n%)	1 (4.3%)	5 (38.4%)	0.048
Milrinone (n%)	6 (26%)	12 (92%)	0.001
Inhaled Nitric oxide (NO)	3 (13%)	9 (69%)	0.0006

*RVSP* Right Ventricular Systolic Pressure, *PDA* Patent Ductus Arteriosus, *PFO* Patent formaen Ovale, *IVS* Interventricular septum

#Vasoactive Ionotropic score(VIS): (dopamine dose (μg/kg/min) + dobutamine dose (μg/kg/min) + 100 × epinephrine dose (μg/kg/min) + 10 × milrinone dose (μg/kg/min) + 10 000 × vasopressin dose (unit/kg/min) + 100 × norepinephrine dose (μg/kg/min)

##### Trends in Echo and Cardiorespiratory Parameters used for Timing Surgical Correction (Fig. 3)

Respiratory severity score (RSS) was high initially in first 72 h, but stabilized in the pre-operative period, and had a modest rise post-operatively.

**Fig. 3:**
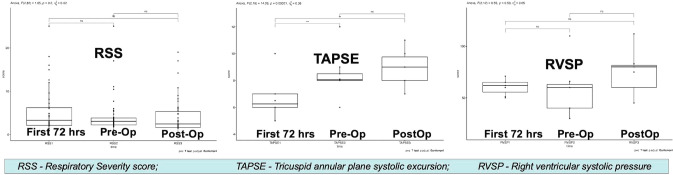
Trended Data of Cardiorespiratory parameters over first three time points

TAPSE was initially low, improved in the pre-op stage and continued to improve post-operatively.

RVSP as a surrogate for pulmonary pressures was elevated in the early 72 h, stabilized pre-operatively and further improved post-operatively.

##### Use of Inotropes, Milrinone, Pulmonary Vasodilators

The frequency of use of milrinone, inotropes and pulmonary vasodilators for our patients with CDH is shown in (Table 5). Milrinone was used in up to 62% of patients, inhaled NO in 59% and sildenafil in 38%.

**Table 5:** Summary of Cardiorespiratory medications

Cardiorespiratory medications used at any time point	No of patients (total = 39)
Inhaled nitric oxide	23 (59%)
Sildenafil (5 started at < 72 h; 1 started at 2wks of age)	15 (38.5%)
Iloprost (one started in first week; other at 44 days of age)	2 (5%)
Milrinone	25 (64%)
Hydrocortisone	9 (23%)
Alprostadil	5 (13%)

## Discussion

This is the first study from Middle Eastern population describing echocardiographic findings in CDH patients. Our calculated estimated prevalence of CDH is 3.82 per 10,000 births, based on average annual births (~ 28,000) in Qatar, [35] as compared to other reported studies quoting prevalence of around ~ 3 per 10,000 births [36]. A total of 42 cases of CDH were admitted to our center over a period of 3.5 years, making it a high-volume center as per international guidelines [37]. The incidence of genetic anomalies in our cohort and in general, in the Middle Eastern populations seems higher, likely due to consanguinity and lower rates of pregnancy termination [38–40]. Although the role of genetics in the pathogenesis of CDH has been well established, only a handful of disease genes have been identified so far. A number of damaging de novo gene variants with potentially pleiotropic effects have also been identified [41].

Our overall mortality is comparable to internationally reported rates (20–40%), but after excluding confirmed genetic anomalies, the mortality was even lower, which is comparable to reports from many high-volume centers [42]. The mortality in preterm infants was double of that seen in term infants, consistent with previous studies reporting similar lower survival rates in preterm infants with CDH [43]. Post-surgical and post-ECMO mortality was also comparable to the CDHSG mortality rate of 14% after surgery [44] and was influenced by several factors, including the timing of surgery and pre-op stability [45, 46]. Need for ECMO in our cohort was significantly low (only 2 patients required ECMO, of which 1 survived) compared to published reports of up to 30% [47, 48]. Echocardiographic assessment was seen to be a vital part of the management approach in these infants, with regards to many of the decisions around inotropes, pulmonary vasodilators, the timing of surgery, and ECMO. We believe that a standardized management [46] approach and the presence of a dedicated hemodynamic team, along with diligent ventilatory support may have contributed to improved survival outcomes, including the avoidance of need for ECMO in several patients.

Pulmonary hypertension is a known predictor of mortality in CDH patients [16]. In our cohort, as many as seventy percent of newborns that died and had early echocardiograms, suggesting moderate to severe PH in the first 72 h. In our cohort**,** RVSP of more than 45.5 in the first 72 h was found to be a good predictor of mortality. Similar cut-off values are reported by Derya et al.[29]. In other reports, the mortality rate was found to be nearly 100% in patients with supra-systemic or systemic pulmonary arterial pressure during the first three weeks of life, but the survival rate in infants with pulmonary arterial pressure below half of the systemic pressure was found to be 100%. The risk of mortality in infants with moderate pulmonary hypertension was 75% [49].

Birth weight less than 2.8 kg was a significant predictor of mortality in our cohort. Previous studies have similarly reported birth weight cut-offs of 2.755 kg as a predictor for mortality [50]. Higher Post-operative VIS and RSS scores were predictive of mortality in our cohort. This is consistent with previous studies reporting on the use of RSS as a predictor of mortality [32] and higher post-op VIS scores being predictive of negative outcomes in infants with CDH [51]. VIS more than 23 was associated with high mortality in our cohort. In patients undergoing cardiac surgery, maximum post-op VIS ≥ 20 predicts an increased likelihood of a poor composite clinical outcome [52]. Our patients in the high VIS group had more severe pulmonary hypertension and cardiac dysfunction. Those with higher RSS had higher pulmonary pressures and higher use of inotropes. This would be consistent with disease severity.

The improved trends pre-operatively for RSS, TAPSE, and RVSP indicate that clinicians endeavored to achieve cardiovascular stability in patients prior to surgery with regards to need for respiratory support, right ventricular systolic function, and pulmonary pressures. Pre-operative high RSS, pulmonary pressures and poor right ventricular function are important factors associated with mortality and morbidity in patients with CDH, and therefore the stability of these parameters is commonly practiced [32, 53, 54]. RSS and pulmonary pressures in the immediate preoperative period were also assessed to be potential predictors but did not achieve statistical significance, likely due to our small numbers. However, generally, surgery was timed after achieving respiratory stability and manageable pulmonary hypertension which resulted in stable post-operative period [50].

Use of Milrinone in our cohort (62%) is higher than in international reports. Milrinone, a phosphodiesterase-III inhibitor with lusitropic and vasodilator effects, was used in up to 30% of CDH infants across the USA. No randomized trials have tested the efficacy or safety of milrinone in CDH neonates, but retrospective studies demonstrated that its use was associated with neither improved OI, PAP, or left ventricular measurements nor adverse events [55]. The use of iNO in our cohort (59%) is comparable to that reported by the Congenital Diaphragmatic Hernia Study Group registry, where the mean percentage of patients treated with iNO by center was 62.3% (range, 0%-100%) [44, 56]. Multiple studies have failed to demonstrate a clear benefit of iNO in CDH infants, but its use is still widespread, and it is considered an essential treatment method in the transitional period of CDH patients [57]. We also had 38% of our patients on sildenafil. Despite the liberal use of these medications in our cohort, our mortality data are comparable or better than some international reports. It is possible that serial and timed functional echocardiography encouraged the timely use of milrinone, pulmonary vasodilators, and appropriate inotropes, which could have played a role in improving our outcomes.

Strengths: Standardised echo schedule and timelines as per protocol were used. The hemodynamic team consists of a group of experienced clinicians. Standardization of technique was emphasized amongst the group by regular hemodynamic meetings and case discussions.

Weaknesses: This is an observational study where stored data were analyzed. Missing images or information could therefore not be obtained retrospectively. Some variation in acquired images and measurements may be present as echocardiograms were performed by several experienced members of the team. In the sicker patients, clinicians prioritized the assessment of only certain important echo parameters, in the interest of maintaining patient stability. Patients with any cardiac anomalies were mainly scanned by cardiologists and therefore did not always have complete functional echocardiography measurements performed at the set time points.

## Conclusions

CDH is a complex, multiorgan disease which has a large spectrum and whose prognosis is not easily predictable. Serial timed functional Echo monitoring allows targeted therapy of patients with CDH. Birth weight, initial pulmonary pressures and postoperative respiratory stability and inotropic scores may be useful predictors of mortality, but further validation is required. Early RSS, pulmonary pressures and right ventricular function are important factors associated with mortality and morbidity in patients with CDH and therefore can be utilized for assessing pre-operative stability and decision-making in timing of surgery. Large prospective clinical studies in CDH patients are needed to identify predictive functional markers and for optimizing management.

## Supplementary Information

Below is the link to the electronic supplementary material.

Supplementary file1 (DOCX 96 kb)

Supplementary file2 (DOCX 484 kb)

Supplementary file3 (MP4 4511 kb)

## Abbreviations

MAP: Mean Airway pressure
RVSP: Right Ventricular Systolic Pressure
PAAT: Peak Acceleration Time
RVET: Right ventricular ejection time
TAPSE: Tricuspid annular plane systolic excursion
VIS: Vasoactive Inotropic score
RSS: Respiratory severity score
f-Echo: Functional echocardiography
PH: Pulmonary hypertension
PPHN: Persistent pulmonary hypertension of newborn
PDA: Patent Ductus Arteriosus
ASD: Atrial septal defect
VSD: Ventricular septal defect
ECMO: Extracorporeal Membrane Oxygenation
