# Supplementary material for: The Utility of Serial Echocardiography Parameters in Management of Newborns with Congenital Diaphragmatic Hernia (CDH) and Predictors of Mortality
Source: Pediatr Cardiol. 2022 Sep 27;44(2):354–66. doi: 10.1007/s00246-022-03002-y (PMC9895036; doi:10.1007/s00246-022-03002-y)
Supplement: Supplementary file 1 — Supplementary file1 (DOCX 96 kb) [file 246_2022_3002_MOESM1_ESM.docx]

**APPENDIX IA: Functional ECHO assessment in CDH**

**APPENDIX IB: Timing of echocardiography in CDH**

| **Timing of**  **f-echo** | Soon after birth, once stable | Before starting inotrope or vasoactive therapy  (0-7) | 24 hours after starting therapy | Daily  (0-7) | Pre-op | 24 hours post CDH repair | Pre ECMO | Every 7th day | Day 14 | Day 28 | Pre discharge | Six months of age | 12 months of age |
| --- | --- | --- | --- | --- | --- | --- | --- | --- | --- | --- | --- | --- | --- |
| **Mandatory** | **X** | **X** | **X** |  | **X** | **X** | **X** |  | **X** | **X** |  | **X** | **X** |
| **Optional** | **X** | **X** | **X** | **X** | **X** | **X** | **X** | **X** | **X** | **X** | **X** | **X** | **X** |
