# Supplementary material for: The Utility of Serial Echocardiography Parameters in Management of Newborns with Congenital Diaphragmatic Hernia (CDH) and Predictors of Mortality
Source: Pediatr Cardiol. 2022 Sep 27;44(2):354–66. doi: 10.1007/s00246-022-03002-y (PMC9895036; doi:10.1007/s00246-022-03002-y)
Supplement: Supplementary file 2 — Supplementary file2 (DOCX 484 kb) [file 246_2022_3002_MOESM2_ESM.docx]

**Appendix I:**

**CDH f-ECHO based management Protocol**

**
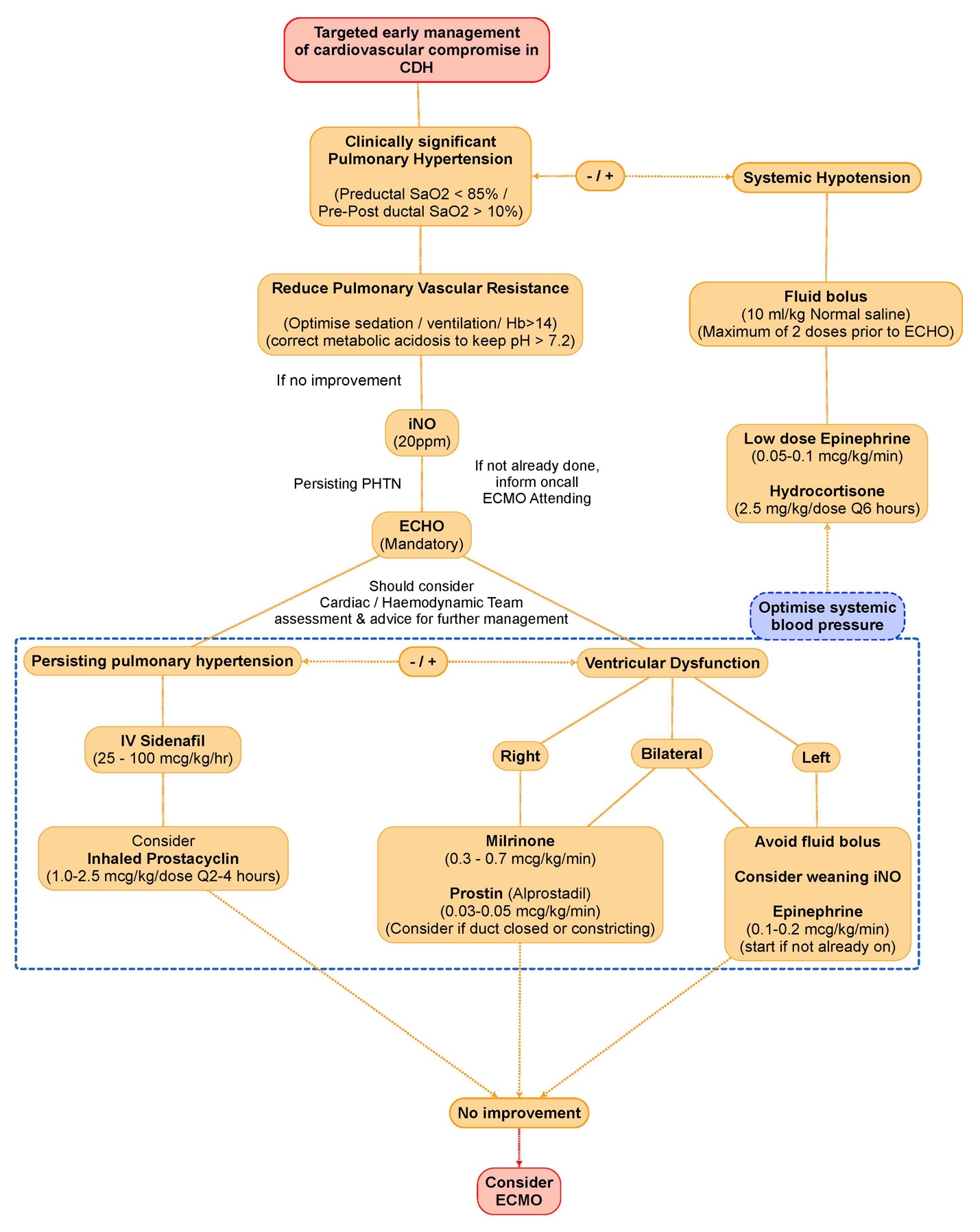
**

Protocol adopted from CODINOS trial^27^ and Glasgow CDH protocol with permission from Dr Neil Patel
